# Analgesia for infants’ circumcision

**DOI:** 10.1186/1824-7288-39-38

**Published:** 2013-06-13

**Authors:** Carlo V Bellieni, Maria G Alagna, Giuseppe Buonocore

**Affiliations:** 1 Department of Paediatrics, Obstetrics and Reproductive Medicine, University of Siena, Policlinico “Santa Maria alle Scotte”, Viale Bracci 2, Siena 53100, Italy

**Keywords:** Male circumcision (MC), Newborn, Analgesia, Pain management

## Abstract

Male circumcision (MC) is one of the oldest and most common operations performed all over the world. It can be performed at different ages, using different surgical techniques, for different religious, cultural and medical reasons.

Our aim is to examine and compare the various methods of analgesia and different surgical procedures reported in literature that are applied in infant MC. We performed a PubMed, MEDLINE, EMBASE and Cochrane search in the papers published since 2000: 14 studies met the inclusion criteria, most of them showing that a combined pharmacological and non-pharmacological intervention is the best analgesic option, in particular when the dorsal penile nerve block is combined with other treatments. The Mogen surgical procedure seems to be the less painful surgical intervention, when compared with Gomco clamp or PlastiBell device. Only 3 papers studied groups of at least 20 babies each with the use of validated pain scales. Data show a dramatic decrease of pain with dorsal penile nerve block, plus acetaminophen associated to oral sucrose or topic analgesic cream. However, no procedure has been found to definetively eliminate pain; the gold standard procedure to make MC totally painfree has not yet been established.

## Introduction

The relief of human suffering is one of the most important goals for health care providers. Advances in neonatology have significantly improved neonatal morbidity and mortality; but pain, discomfort, and stress remain sad realities for babies in the neonatal intensive care unit [1]. Assessing, managing, and trying to limit these clinical realities, particularly while caring for neonates are challenging and increasingly controversial [2]. Newborns’ pain can harm the developing brain in several ways, among which is the increase of free radical production [3].

Male circumcision (MC) is one of the most painful procedures a newborn can undergo, but only in the last few years caregivers have tried to fight this kind of pain; this might be due to the pain being in some ways, a component of the ritual that for centuries has accompanied MC. Unfortunately, even during clinical trials, babies still undergo MC without analgesia [4] and the continuous production of studies for a better analgesia is the sign that a gold standard has not yet been found.

MC consists of the surgical removal of the sleeve of skin and mucosal tissue which normally covers the glans of the penis, known as the foreskin. The word ‘circumcision’ derives from the Latin circum (meaning ‘around’) and caedere (meaning ‘to cut’) [5].

For many centuries, MC has incited great fervour in opposing parties who have debated whether the medical benefits of the procedure outweigh any potential psychological side-effects resulting from it. About 30% of the total world male population is circumcised and MC remains one of the oldest and most common operations performed all over the world [6,7]. It is one of the oldest surgical operations, with the earliest available records dating this ancient procedure back to at least 6000 years BC, and anecdotal evidence suggesting it as a rite of puberty in aboriginal tribes before 10000 BC [8].

MC is commonly conducted for religious, cultural and medical reasons; it can be performed at different ages, in neonates, infants and children, with important differences in complication rates. Neonatal MC seems to be a simple, quick procedure, healing within 1 week with a low rate of usually minor adverse events (0.2%–0.4% in the US) when performed in clinical settings by trained professionals [9]. There is a high rate of circumcision in Jewish and Muslim populations, and circumcision is quite common in the United States. Areas of Africa, Australian aborigines, and people of Eastern America also practice ritual MC. In contrast, routine MC was rarely performed in Europe, China and Central and South America, but the incidence is currently increasing due to migration [10]. Traditionally, the US medical establishment promoted MC as a preventative measure for an array of pathologies including reduced risks of penile cancer, urinary tract infections, sexually transmitted diseases, and even cervical cancer in sexual partners [11].

The three most common operative methods of MC for the newborn male include: the PlastiBell device, the Gomco clamp and the Mogen clamp [12-16]. All techniques cause similar amounts of tissue destruction [16].

The aim of this review is to examine and summarize all studies in literature since the year 2000 that have compared various methods of analgesia during newborn/infant MC.

## Methods

We performed a PubMed, MEDLINE, EMBASE and Cochrane search in studies published in the last 12 years using the following as keywords and MeSH terms: pain, anaesthesia/analgesia, infant, newborn, pediatric and male circumcision. We included studies in which the mean age at MC was age 11 months or less. Studies were included in our research if they compared different types of anaesthesia/analgesia or different surgical techiniques, and if they used specific pain scales.

## Results

Among a total of 77 papers found, published to 2000, only 14 (Table 1) of these met the following inclusion criteria: comparison in relation to various analgesic methods or different surgical procedures and evaluation of newborns’ pain assessment, using specific or non-specific pain scales.

**Table 1:** Papers meeting the inclusion criteria, collected in chronological order

**Authors article**	**Newborns**	**Analgesia**	**Surgical technique**	**Pain scale**	**Results**	**Pub date**
O’Sullivan et al. [17]	66	DPNB (ultrasound or anatomical landmark) / fentanyl	ukn*	FLACC	No significant difference between the groups in terms of fentanyl administration. The ultrasound technique took longer to perform but suggests some benefits in terms of postoperative pain.	2011
Banieghbal et al. [18]	583	RB/milk	Gomco	NIPS	RB causes a painful response in the majority of the babies, but this is mostly noted in babies over one week of age.	2009
Lehr et al. [19]	44	DPNB/EMLA/lidocaine	Gomco	NFCS/crying time	No significant difference in analgesic efficacy between treatments.	2007
Garry et al. [20]	18	DPNB/EMLA	ukn*	NIPS/numeric pain scale	DPNB is significantly more effective for pain relief than topical EMLA.	2006
Lehr et al. [21]	53	DPNB/EMLA/lidocaine	Gomco	Heart rate/respiratory rate/SpO_2_	No significant difference in analgesic efficacy between treatments.	2005
South et al. [22]	44	DPNB/Tylenol/non-nutritive sucking	Gomco	PIPP/crying time/ salivary cortisol level	NNS significantly decreases the pain response, in addition to other common analgesics.	2005
Razmus et al. [23]	132	DPNB/DPNB+sucrose/ DPNB+sucrose+EMLA/ EMLA/ EMLA+sucrose/ RB/ RB+sucrose solution/ sucrose solution	ukn*	FLACC	DPNB and RB in combination with the concentrated oral sucrose have the lowest pain scores. The sucrose alone does not provide sufficient analgesia; however, it can reduce the pain scores somewhat when used in conjunction with other analgesics.	2004
Malnory et al. [24]	53	Acetaminophen	Gomco	NIPS	Lower pain scores in newborns who received analgesia than placebo.	2003
Taeusch et al. [25]	59	DPNB/dextrose solution	Mogen vs PlastiBell	Cry response	The Mogen technique is preferred over the PlastiBell because of the simplicity of execution, and it is also associated with less pain and discomfort and it takes less time.	2002
Kaufman et al. [26]	57	EMLA/sucrose solution	Mogen vs Gomco	Crying time/ facial grimancing	Mogen appears a better procedure than Gomco, and it takes less time, showing also that the use of analgesia is imperative.	2002
Macke et al. [27]	60	Acetaminophen	Gomco	NCAFS/crying time/heart rate	No significant difference in analgesic efficacy between treatments and placebo.	2001
Joyce et al. [28]	23	EMLA/music	ukn*	RIPS/heart rate/SpO_2_/salivary cortisol level/crying time	Efficacy of EMLA and music to contribute to the pain relief of neonates undergoing circumcision.	2001
Kass et al. [29]	71	DPNB/dextrose solution	Gomco	MBPS/ crying time/heart rate/respiratory rate	No significant differences between the oral glucose and water groups among any of the pain-related measurements; only the use of DPNB shows significantly lower pain scores and reduced objective measurements of pain and physiologic stress.	2001
Taddio et al. [30]	86	DPNB/EMLA/acetaminophen	Mogen vs Gomco	NFCS/crying time	Infants circumcised with the Mogen clamp and combined analgesia have also less pain than those circumcised with the Gomco clamp and EMLA cream, and it also takes less time.	2000

*No data was available regarding the specific surgical procedure to perform MC.

Abbreviations: *NIPS* Neonatal infant pain scale, *RIPS* Riley infant pain scale, *PIPP* Premature infant pain profile, *MBPS* Modified behavioral pain scale, *NFCS* neonatal face coding system, *NCAFS* nursing child assessment feeding scale, *DPNB* Dorsal penile nerve block, *RB* Ring block.

In the papers, that fulfilled the inclusion criteria, different surgical procedures were used: Gomco clamp, Mogen clamp and PlastiBell device [12-16], explained and summarized in Table 2.

**Table 2:** Surgical procedures of MC

**PlastiBell device**	It is the application of a bell-shaped plastic shield over the penis glans and the tying of a tight ligature around the bell and foreskin prior to the amputation; this limits bleeding and produces ischaemic necrosis of the residual foreskin stump, avoiding the need for sutures [12,13].
**Gomco clamp**	The foreskin is retracted, congenital preputial adhesions are separated, and the appropriate-sized bell of the clamp is placed over the glans. The foreskin is replaced over the bell, and the clamp is assembled. Closure of the clamp crushes the skin, allowing the distal prepuce to be excised and producing a suture-less anastomosis just below the corona [12].
**Mogen clamp**	It is Jewish ritual MC; this has a slit through which the foreskin is pulled and then crushed above the glans; the distal foreskin is excised before the inner prepuce is retracted, and a circumferential dressing is applied [12].

Table 3 shows the papers [17-30] that compared different analgesic methods.

**Table 3:** Papers that compared different analgesic methods

**Authors**	**Newborns (n)**	**Surgical technique**	**Analgesia**
Banieghbal et al. [18]	583	Gomco	**RB**/ RB+milk*
Lehr et al. [21]	53	Gomco	DPNB (n=18) /EMLA (n=17) /lidocaine (n=18)
Lehr et al. [19]	44	Gomco	DPNB (n=17) /EMLA (n=13) /lidocaine (n=14)
South et al. [22]	44	Gomco	DPNB + Tylenol (n=22) /**DPNB+Tylenol+non-nutritive sucking** (n=22)
Malnory et al. [24]	53	Gomco	**Acetaminophen** (n=26) vs placebo (n=27)
Macke et al. [27]	60	Gomco	Acetaminophen (n=29) vs placebo (n=31)
Kass et al. [29]	71	Gomco	**DPNB** (n=24) /dextrose solution (n=23)
O’Sullivan et al. [17]	66	unknown	**DPNB**/fentanyl*
Garry et al. [20]	18	unknown	**DPNB** (n=6) /EMLA (n=6)/ no analgesia (n=6)
Razmus et al. [23]	132	unknown	DPNB (n=7) /**DPNB+sucrose solution** (n=12) / DPNB+sucrose solution+EMLA (n=3) / EMLA(n=6) /EMLA+sucrose solution (n=8) / RB (n=15) / **RB+sucrose solution** (n=44) / sucrose solution (n=22)/ no analgesia (n=11)
Joyce et al. [28]	23	unknown	EMLA (n=11) / **EMLA+music** (n=12)

In bold, the most effective analgesic method. In brackets, the number of babies in each study-group.

*No data was available regarding the number of babies in each study-group.

Abbreviations: *RB* Ring block, *DPNB* Dorsal penile nerve block.

Main pharmacological strategies were:

EMLA cream: eutectic mixture of local anesthetics, with 2.5% lidocaine and 2.5% prilocaine, that produces dermal analgesia, applied as a topical cream to the distal half of the penis beneath an occlusive dressing 60–90 minutes before the procedure [31];

dorsal penile nerve block (DPNB): regional anaesthesia often obtained with 0.4 ml of 1% lidocaine injected into the fascia beneath the base of the penis at the 10:00 and 2:00 positions using a 27-gauge needle [14,32];

subcutaneous penile ring block (RB): 0.8 ml of 1% lidocaine without epinephrine, injected in a circumferential ring around either the midshaft or at the level of the corona [14,33,34].

Other pharmacological interventions used were: acetaminophen, lidocaine cream, fentanyl, tylenol; non-pharmacological measures were: breast milk, 20% sucrose solution, 50% dextrose solution, non-nutritive sucking (NNS), audio-stimulation with music [32-36].

Seven papers [17,19-23,29] considered DPNB, five of which showed the efficacy of DPNB [17,20,22,23,29] as preoperative analgesia: one of these considered it in combination with NNS and tylenol [22], and one showed the use of DPNB associated to RB and sucrose solution to be more effective [23]. Five studies compared the use of EMLA with other analgesics [19-21,23,28], only one [28] showed its analgesic effect, especially in combination to music. Two papers [24,27] compared acetaminophen with placebo, but they gave contrasting results. Two studies [18,23] showed the efficacy of RB, one [23] in association with DPNB and oral sucrose. Two papers [19,21] evaluated the use of lidocaine cream, but both did not find any analgesic efficacy. One study considered the use of milk [18], one evaluated the use of dextrose solution [29] and one considered the use of fentanyl [17], but none of these had proved effective to decrease the pain response. Seven articles [18,19,21,22,24,27,29] analyzed analgesic treatments when using the Gomco technique. Two papers [19,21] underlined that there was no significant analgesic difference between DPNB, EMLA and lidocaine cream; one of them showed the effectiveness of NNS, in combination to tylenol and DPNB, to decrease the pain response [22]; one paper showed the efficacy of DPNB compared with oral dextrose [29]; one study showed the RB utility as preoperative analgesic [18], and two papers [24,27] gave no univocal conclusions in the case of acetaminophen: one [27] excluded any analgesic effect, compared to placebo, but another paper [24] showed its analgesic efficacy. Four papers [17,20,23,28] did not disclose which surgical procedure was used. One showed the effectiveness of EMLA cream in association with music to decrease the pain response [28]; one paper showed that DPNB was more effective than EMLA cream [20]; one study showed major analgesic effect using oral sucrose in combination with other common analgesics, especially with RB and DPNB at the same time [23]; one paper suggested the use of ultrasound DPNB because it was associated with a reduction in terms of analgesic postoperative requirement [17].

Only three papers [25,26,30] compared different surgical techniques to perform MC, as shown in Table 4; all three studies found a greater analgesic effectiveness of Mogen clamp than both Gomco and PlastiBell. In particular, two of these papers [26,30] compared Mogen with Gomco clamp and both found a best analgesic effect of Mogen in terms of performing time and to decrease the pain response, if associated with preoperative analgesia, especially using a combination of analgesics (DPNB with EMLA and acetaminophen [30], and EMLA with sucrose solution [26]). One paper compared the use of the Mogen clamp with the Plastibell device [25], showing that the Mogen procedure is associated with less pain, stress and discomfort.

**Table 4:** Papers that compared different surgical procedures to perform MC

**Authors**	**Newborns (n)**	**Mogen**	**Gomco**	**Plastibell**	**Concurrent analgesia**	**Pain scale**
Taeusch et al. [25]	59	**30**	-	29	**DPNB + dextrose solution** (all babies)	Cry response
Kaufman et al. [26]	57	**29**	28	-	**EMLA+sucrose solution** (Gomco n=14, **Mogen n=14**) /EMLA+water (Gomco n=14, **Mogen n=15**)	Crying time/facial grimancing
Taddio et al. [30]	86	**57**	29	-	**DPNB+EMLA+ acetaminophen (Mogen)** / EMLA (Gomco)	NFCS/ crying time

In bold, the most effective analgesic method or the least painful surgical procedure.

Abbreviation: *DPNB* Dorsal penile nerve block.

Only 7 papers enrolled twenty or more babies for each study-group [22,24-27,29,30].

Table 5 shows the papers in which twenty or more babies were enrolled in each study-group and used validated pain scales and respective pain scale scores, with the data available [22,24,30].

**Table 5:** Comparison among the papers that had twenty or more babies in each study-group and simultaneously used the specific pain scale and respective pain scale scores available

**Authors**	**Newborns (n)**	**Analgesia**	**Specific pain scale**	**Pain scale scores**
Malnory et al. [24]	53	**Acetaminophen**	NIPS (0–7)	1.55 (1.19-2.25)
		placebo		2.55 (2.1-3.0)
South et al. [22]	44	**DPNB+acetaminophen+non-nutritive sucking**	PIPP (0–21)	5.7 (3.7-7.0)
		DPNB+Tylenol		8 (6.8-8.5)
Taddio et al. [30]	86	**DPNB+EMLA+acetaminophen**	NFCS (0-100%)	30% (20-40%)
		EMLA		95% (93-97%)

In bold, the most effective analgesic method.

In Table 6 we report the rates of acute and long-term adverse events occurring in neonatal MC, as reported in literature [30,37-44]. As shown there, the Gomco clamp and the Plastibell device are associated to complications, including acute complications, such as bleeding or infection, or long-term adverse events, such as adhesions and meatal stenosis. No data about complications of the Mogen technique are available, and are reported as “rare” [37].

**Table 6:** Frequency of acute and long-term adverse events in neonatal circumcision

**Surgical technique**	**Short and long-term possible risks**	**Frequency (%) of adverse events**
GOMCO clamp	- severe infection requiring antibiotics;	0.3%-15%
	- severe meatal ulcer;	
	- urethral laceration;	
	- bleeding;	
	- meatal stenosis;	
	- foreskin adhesions;	
	- meatitis;	
	- requiring circumcision revision [30,37-44].	
**MOGEN clamp**	**- Need of repeating the procedure if the penis size is small**[30,37-44]	**Not available, but reported as “rare”**
PLASTIBELL device	- severe infection requiring antibiotics;	0%-8%
	- severe meatal ulcer;	
	- Plastibell ring device itself, which is left on after the procedure and normally takes 7–10 days to fall off. The problems included delayed separation of the ring, incomplete separation of the ring, or the ring becoming stuck on the penile shaft;	
	- foreskin adhesions;	
	- meatitis;	
	- requiring circumcision revision [30,37-44].	

## Discussion

Our data discloses large heterogeneity with regard to size of the samples, pain scales, combinations of pharmacological and non-pharmacological analgesia and surgical techniques. Some studies did not specify the MC procedure applied, and others used non-specific infants’ pain assessments (e.g. crying time, heart rate, respiratory rate). Therefore, it was not possible to compare the data of the different studies because only 7 papers enrolled twenty or more babies for each study-group [22,24-27,29,30]. Only three papers [22,24,30] fulfilled both the conditions of enrolling this number of babies and of using a validated and specific pain scale. The use of a validated pain scale is important because other ways of assessment of pain (e.g. crying time) are neither specific nor sensitive enough.

Among three papers [22,24,30] collected in Table 5, no authors compared the same analgesic methods using the same pain scale; whereby, it is impossible to compare them to make a meta-analysis. This selected data shows that the use of DPNB, in association to tylenol and NNS [22] and DPNB in association to EMLA and acetaminophen [30] reduce the pain response, but not totally. Acetaminophen [24] seems to be more effective (its main pain score compared with the upper limit of the scale is very low), but it is surprising that the main pain scale score is so low using placebo [24].

In particular, DPNB appears to be more effective in association with tylenol and NNS [22] and in combination with RB and oral sucrose solution [23]. Moreover, the Mogen clamp surgical procedure seems to be less painful than the other techniques [25,26,30]. This data confirms the previous analysis of data available to 2001 [45], when DPNB was shown to be the most effective analgesic method. Our data is similar to that of 2001 [45] also with regard to the effectiveness of the Mogen clamp.

We have reported in Table 6 the clinical drawbacks of the different surgical techniques: the Mogen techniques seems to be the safest. In Table 5 we reported the level of pain in the most reliable studies we retrieved. No study guarantees a complete analgesia, with the exception of one [24], whose limits we have previously described.

Recently, a non-pharmacological technique used for the relief of pain both in term and preterm infants, called “Sensorial Saturation” (SS) has been proposed and validated. It consists in attracting the baby’s attention with positive stimuli (tactile, auditory, gustatory and visual), so as to reduce up to nullify the perception of painful stimuli. This technique is based on neuro-physiological concepts, according to which the newborn's brain is able to “filter” the peripheral stimuli through the “gate control system”. In this way, the above stimuli “saturate” the central receptors, resulting in a “sensorial jam” that excludes painful stimuli. Several studies [46,47] show the effectiveness of the SS, also used in national and international analgesia protocols. SS is not only a "technique", but a way of being with the child, involving parents and making them the protagonists of the medical event. We propose (Table 7) a new analgesic approach for infantile MC using SS in association with DPNB.

**Table 7:** Five steps for the analgesia for neonatal MC

**1-**	Contain the baby in a calm environment
**2-**	Talk to the baby to attract his/her attention
**3-**	Massage his/her face, and give some drops of sweet solution on the tongue to obtain a regular sucking
**4-**	When the baby has achieved a regular suction, perform DPNB
**5-**	Perform MC using the Mogen technique; meanwhile, continue to stimulate the baby throughout the procedure

You can apply EMLA cream on the skin 60 minutes before the procedure.

Abbreviations: *DPNB* Dorsal penile nerve block, *MC*: male circumcision.

In conclusion, more research is required to find a better analgesic approach, in order to make infantile MC a totally painless procedure without stress or discomfort for newborns. Present methods do not yet guarantee a total analgesia during this procedure.

## Competing interests

The authors declare that they have no competing interests.

## Authors’ contributions

CVB and GA performed the analysis of the literature. GB collaborated in the discussion. All authors read and approved the final manuscript.
